# Advanced neonatal procedural skills: a simulation-based workshop: impact and skill decay

**DOI:** 10.1186/s12909-023-04000-1

**Published:** 2023-01-13

**Authors:** Amelie Stritzke, Prashanth Murthy, Elsa Fiedrich, Michael-Andrew Assaad, Alexandra Howlett, Adam Cheng, David Vickers, Harish Amin

**Affiliations:** 1 Section of Neonatology, Department of Pediatrics, Cumming School of Medicine, University of Calgary, Libin Cardiovascular Institute of Alberta, Alberta Children’s Hospital Research Institute, Foothills Medical Centre, 780-1403 29Th St NW, Calgary, AB T2N 2T9 Canada; 2 Alberta Health Services, Calgary, Canada; 3 Libin Cardiovascular Institute of Alberta, Calgary, Canada; 4 University of Montreal, Quebec, Canada; 5 Simulation Program, Alberta Children’s Hospital, Calgary, Canada; 6 Mozell Core Analysis Lab, Libin Cardiovascular Institute of Alberta, Calgary, Canada

**Keywords:** Simulation, Skill decay, Procedures, Neonatal procedures, Workshop

## Abstract

**Background:**

Trainees aiming to specialize in Neonatal Perinatal Medicine (NPM), must be competent in a wide range of procedural skills as per the Royal College of Canada. While common neonatal procedures are frequent in daily clinical practice with opportunity to acquire competence, there are substantial gaps in the acquisition of advanced neonatal procedural skills. With the advent of competency by design into NPM training, simulation offers a unique opportunity to acquire, practice and teach potentially life-saving procedural skills. Little is known on the effect of simulation training on different areas of competence, and on skill decay.

**Methods:**

We designed a unique simulation-based 4-h workshop covering 6 advanced procedures chosen because of their rarity yet life-saving effect: chest tube insertion, defibrillation, exchange transfusion, intra-osseus (IO) access, ultrasound-guided paracentesis and pericardiocentesis. Direct observation of procedural skills (DOPS), self-perceived competence, comfort level and cognitive knowledge were measured before (1), directly after (2), for the same participants after 9–12 months (skill decay, 3), and directly after a second workshop (4) in a group of NPM and senior general pediatric volunteers.

**Results:**

The DOPS for all six procedures combined for 23 participants increased from 3.83 to 4.59. Steepest DOPS increase pre versus post first workshop were seen for Defibrillation and chest tube insertion. Skill decay was evident for all procedures with largest decrease for Exchange Transfusion, followed by Pericardiocentesis, Defibrillation and Chest Tube. Self-perceived competence, comfort and cognitive knowledge increased for all six procedures over the four time points. Exchange Transfusion stood out without DOPS increase, largest skill decay and minimal impact on self-assessed competence and comfort. All skills were judged as better by the preceptor, compared to self-assessments.

**Conclusions:**

The simulation-based intervention advanced procedural skills day increased preceptor-assessed directly observed procedural skills for all skills examined, except exchange transfusion. Skill decay affected these skills after 9–12 months. Chest tube insertions and Defibrillations may benefit from reminder sessions, Pericardiocentesis may suffice by teaching once. Trainees’ observed skills were better than their own assessment. The effect of a booster session was less than the first intervention, but the final scores were higher than pre-intervention.

**Trial Registration:**

Not applicable, not a health care intervention.

**Supplementary Information:**

The online version contains supplementary material available at 10.1186/s12909-023-04000-1.

## Background

Pediatric residents lack key resuscitative and critical life-saving skills at the end of their training [1, 2]. Only 17% were confident enough to independently conduct neonatal resuscitations, despite the Royal College of Canada (RCPSC) mandated pediatric competencies at the end of training [3–5]. Neonatal Perinatal Medicine (NPM) subspecialty trainees (pediatricians seeking specialized training) must be competent in an even wider range of procedural skills [6]. While NPM trainees perform better than junior residents in common neonatal procedures, there are still substantial gaps in the performance of more advanced procedural skills [7, 8]. With a documented reduction in clinical training opportunities, [9, 10] and with the transition to competency-by-design (CBD) in Canadian pediatric training, many objectives of training cannot be met by opportunistic clinical experience alone [11, 12].

Performance of infrequent procedural skills are addressable via simulation practice [13]. By utilizing adult learning theory principles such as self-direction, frequent feedback, and Ericsson’s principle of deliberate practice [14], simulation is the promising method for procedural learning [15, 16]. Simulation-based procedural training has been shown to be effective at achieving positive learner outcomes with regards to behavior and skill training [17], help reduce procedure complications, [18] uncover more critical errors than written examination [19], and has a role for life-long skill maintenance [20].

There is limited data on the effectiveness of simulation-based training for critical neonatal procedural skills. Addressing this gap will help inform training methodologies for neonatal trainees, with the ultimate goal of improving procedural competence and patient outcomes. In this study, we aim to assess the impact of an educational intervention targeting six rarely performed procedures in a cohort of NPM and senior general pediatrics trainees on observed skill level in simulation, self-perceived competence, comfort, and cognitive knowledge. We will describe skill acquisition and retention by measuring outcomes immediately before and after training, and at long-term follow-up.

## Methods

### Study design and participants

We conducted a prospective observational cohort study with a pre-post design. NPM subspecialty trainees and senior pediatric residents enrolled at Cumming School of Medicine, Calgary, Canada, were approached to participate. Background training information was collected, International Medical Graduate (IMG), or Canadian Medical Graduate (CMG), and year of training. This constitutes an opportunistic convenience sample.

Inclusion criteria were NPM trainees, or senior pediatric residents (PGY-3 or -4), and willingness to participate in 2 required sessions, 9–12 months apart. There were no specific criteria for exclusion. Written informed consent was obtained from all participants. Conjoint Health Research Ethics Board (CHREB) approval was granted (REB18-1079).

### Recruitment

Calgary pediatric residency and NPM subspecialty training Program Directors approved this study. An email invitation to all eligible trainees was sent and all interested individuals were accommodated. Participation in this research study was voluntary, and no feedback occurred between preceptors and Program Directors as to individual performances.

### Intervention

Six critical procedures were identified as skills requiring further training (low self-assessed competence scores) based on a prior cross-sectional survey of 47 NPM trainees from 13 Canadian programs (unpublished data). “Advanced Neonatal Procedural Skills Day” workshops of 4 h duration each were offered during 2018–2020, as in-person simulation-based practice of the six selected skills. The workshop was developed by *AS*, following Kern’s approach for curriculum development [21]. Input was sought from neonatal nursing educators, pre-existing educational materials and local practice guidelines. Further video instructions and review articles were added to Course Materials. For both the Paracentesis and Pericardiocentesis stations, specific task trainers were developed locally (Appendix A). Course Materials were sent 2 weeks in advance in a flipped classroom approach, after obtaining consent for the research study [22, 23].

- Welcome letter (Appendix B) with course overview, agenda, timeline (Appendix C), and instructions on how to do the surveys before pre-course videos and materials are viewed.
- SurveyMonkey™ links with questionnaires regarding self-perceived competence, comfort level, and cognitive knowledge regarding each procedure.
- Pre-Course video links, to be viewed after initial survey, and before attending course.

“Advanced Neonatal Procedural Skills Day”: Six stations were run, each 40 min long with 5 min intermission for 1–2 participants each (Table 1). Each station was taught by 1–2 preceptors who are experts in their fields and who had reviewed the pertinent course materials (Table 1). Consistency in preceptors ensured consistent delivery of content between workshops.

**Table 1:** Skill stations for Advanced Neonatal Procedural Skills Day

Number	Skill	Teacher	Task Trainer	Task
1	**Chest Tube**	Neonatologist	Chicken	Insertion of chest drainage tube via Trochar method
2	**Defibrillation**	PALS^a^ Nurse Educators	Rhythm Generator	Recognition of shockable rhythm and appropriate voltage and procedure applied
3	**Exchange Transfusion**	NICU^b^ Nurse Educator And neonatologist	Double UVC/UAC access set-up on mannikin	Calculating blood volume required. Confirming appropriate access and set-up. Withdrawal of blood aliquots from venous access corresponding to concomitant arterial blood transfusion via pump
4	**Intra-osseus access**	Neonatologist	Artificial bones, drills	Use of drill to insert access to appropriate location
5	**Paracentesis**^**c**^	Neonatologist with ultrasound skills	Locally designed task trainer	Selection of most appropriate and safest location under ultrasound-guidance and needle insertion/fluid withdrawal
6	**Pericardiocentesis**^**c**^	Neonatologist with ultrasound skills	Locally designed task trainer	Safest needle entry point, needle insertion/fluid withdrawal under ultrasound-guidance

^a^Pediatric Advanced Life Support

^b^Neonatal Intensive Care Unit

^c^Appendix A: Locally designed task trainers

Pre-assessment: Each participant was assessed individually before teaching and together as a group to learn from each other. Each trainee was asked to briefly review indications, contraindications, and potential complications for each of the individual procedures, and perform the procedure under observation, if the participant felt competent.

Individual teaching: Depending on learners’ needs, not following a specific script, coaching included discussion and correction of any mistakes. The procedure was either shown or coached through, with deliberate practice. Repeated attempts within the time frame were encouraged until both participant and trainer were satisfied. The final procedure without further help was scored as post-assessment. A summary of learning points for most of the procedural skills was sent out to participants after the workshop for easy reference (Appendix D).

Outcomes and Assessment Tools: Primary outcome was: (a) Preceptor assessment of competence for each of the skills before (Time point 1) and after (Time point 2) simulation training “Advanced Neonatal Procedural Skills Day” via Direct Observation of Procedural Skill (DOPS, Appendix E) tool adopted from Barton [24].

Secondary outcomes: a) Self-perceived competence, comfort level, and cognitive knowledge for each of the advanced neonatal procedures before (Time point 1) and after (Time point 2) simulation training “Advanced Neonatal Procedural Skills Day”; and (b) Assessment of DOPS, self-perceived competence, comfort level, and cognitive knowledge for each of the skills 9–12 months after (Time point 3) simulation training which represents skill decay, and after a second intervention (Time point 4). c) Any DOPS difference between CMGs and IMGs.

The Direct Observation of Procedural Skills (DOPS) tool was first described by Wilkinson, [25] and adapted by Barton for the UK Foundations Doctors’ Program (Appendix E). Scored items are based on features of procedural skills such as consent, preparation, technical ability and awareness of limitations and complications. Barton showed its validity and reliability of 0.81 for trainees learning colonoscopies, and that scores were highly correlated with global assessments [24]. The entrustability scale used for each of the items on the DOPS was adapted from Gofton [26] and since also used by the RCPSC adaptation of CBD [4, 6]. Preceptors were given their station’s DOPS to familiarize themselves, but no specific training or calibration was conducted. Rating occurred in real time and was not blinded.

Self-perceived competence and comfort level to perform each procedure if clinically indicated was assessed on a Likert scale from 1–5 with 1 (uncomfortable) and 5 (comfortable) [27]. Similar tools have been used to correlate self-efficacy of NICU health care providers with performance of chest compressions and ventilation on a manikin [28]. Self-perceived competence is relevant to correlate with objective performance data, and comfort level was used to assess whether participants would be willing to attempt the procedure faced in a real patient.

Cognitive knowledge was assessed via survey questions for each procedure, developed by *AS*. These were chosen based on RCPSC procedural knowledge of indications, contraindications, and possible complications. An answer key was provided a priori based on literature review [4]. Scores were given by a single evaluator (*EF*) with 0–1 points for each indication, contraindication and potential complication for a max score of 3 for each procedure.

### Analysis

Descriptive statistical analysis was used. Mean, median and standard deviations were used for the continuous variables and evaluations. We used R [29] and the package “lme4” [30] to perform a repeated measures analysis of the relationship between the score from each assessment tool and the four study time points. We used time point as out single into the model, and as random effects we included intercepts for participants and procedural skill. Using participant and procedure as random effects accounts for two sources of non-independence. Any observable effect over time was quantified through the slope term for each skill.

## Results

There were six “Advanced Neonatal Procedural Skills Days” conducted between August 2018 and September 2020 (Aug 22, 2018; July 3, July 17, and Sept 4, 2019; July 15 and Sept 9, 2020) with 23 trainee participants (16 NPM trainees and 7 pediatric residents), 16 (70%) of which completed two workshops (11 NPM traineess and 5 residents). Reasons not to attend the second workshop for the 7 dropouts were: finished the program and moved away (× 3), maternity leave (× 2), dates could not be accommodated (× 1), and dropping out of the program (× 1). There were 15 IMGs and 8 CMGs, one CMG was an NPM trainee. Out of the 16 NPM trainees, 9 were 1^st^ year, 5 were 2^nd^ year, and 2 were in their 3^rd^ year.

**Fig. 1:**
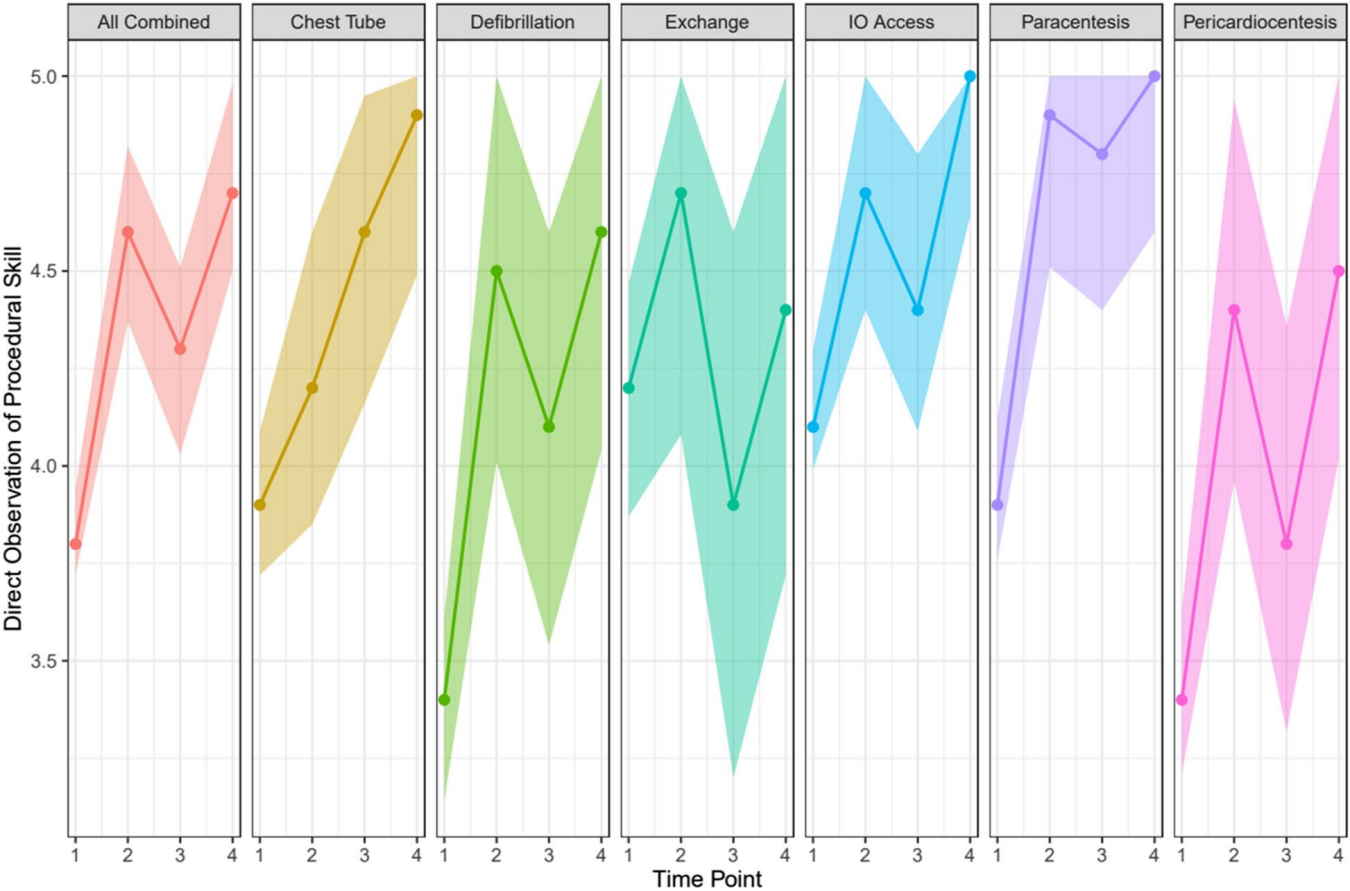
DOPS (Direct Observation of Procedural Skill) changes across time points (shaded area 95% CI)

The DOPS for all six procedures combined increased from 3.83 (95% CL 3.72–3.94) to 4.59 (95% CL 4.37–4.82) post first intervention and to 4.74 (95% CL 4.5–4.98) after second intervention. Figure 1 shows the changes for each of the 6 procedures over the four time points: Steepest slopes with most skill increases were seen for Defibrillation and Chest Tube insertion. The only skill with no significant change was Exchange Transfusion (Fig. 1). When comparing CMGs vs IMGs for each of the skills, CMGs were consistently better in Defibrillation only, while IMGs exceeded, or were equal in all the other skills (data not shown).

Skill Decay: There was evidence of skill decay in the DOPS comparisons for all procedures: The largest decrease was shown for Exchange Transfusion, followed by Pericardiocentesis, Defibrillation and Chest Tube (Table 2). There was little drop in IO and Paracentesis. Self-perceived competence and comfort in contrast increased most for Chest Tube, followed by Defibrillation (Table 2). Worsening self-perceived competence were noted for Exchange Transfusion while comfort increased for all (Table 2).

**Table 2:** Comparing Skill Decay between post-intervention Year 1 and Pre-intervention Year 2 (Time Points 2 and 3)

	**DOPS**		**Self-perceived Competence**		**Comfort**	
**Skill**	**Post-Yr. 1 (95% CI)**	**Pre-Yr. 2 (95% CI)**	**Post-Yr. 1 (95% CI)**	**Pre-Yr. 2 (95% CI)**	**Post-Yr. 1 (95% CI)**	**Pre-Yr. 2 (95% CI)**
Chest Tube	4.2 (3.9, 4.6)	4.6 (4.2, 4.9)	4.0 (3.2, 4.8)	4.5 (3.6, 5.0)	4.3 (3.6, 4.9)	4.8 (4.1, 5.0)
Defibrillation	4.5 (4.0, 5.0)	4.1 (3.5, 4.6)	3.8 (2.9, 4.7)	4.1 (3.1, 5.0)	4.2 (3.4, 5.0)	4.5 (3.6, 5.0)
Exchange Transfusion	4.7 (4.1, 5.4)	3.9 (3.2, 4.6)	3.8 (2.8, 4.8)	3.5 (2.5, 4.6)	4.1 (3.4, 4.9)	4.3 (3.4, 5.0)
IO Access	4.7 (4.4, 5.0)	4.5 (4.1, 4.8)	4.1 (3.4, 4.9)	4.3 (3.4, 5.0)	4.5 (3.7, 5.0)	4.8 (4.0, 5.0)
Paracentesis	4.9 (4.5, 5.0)	4.8 (4.4, 5.0)	3.7 (2.7, 4.7)	3.9 (2.9, 5.0)	4.1 (3.2, 5.0)	4.6 (3.6, 5.0)
Pericardiocentesis	4.5 (3.9, 4.9)	3.8 (3.3, 4.4)	2.9 (2.1, 3.7)	2.9 (2.0, 3.8)	3.4 (2.5, 4.3)	3.9 (2.9, 4.8)

**Fig. 2:**
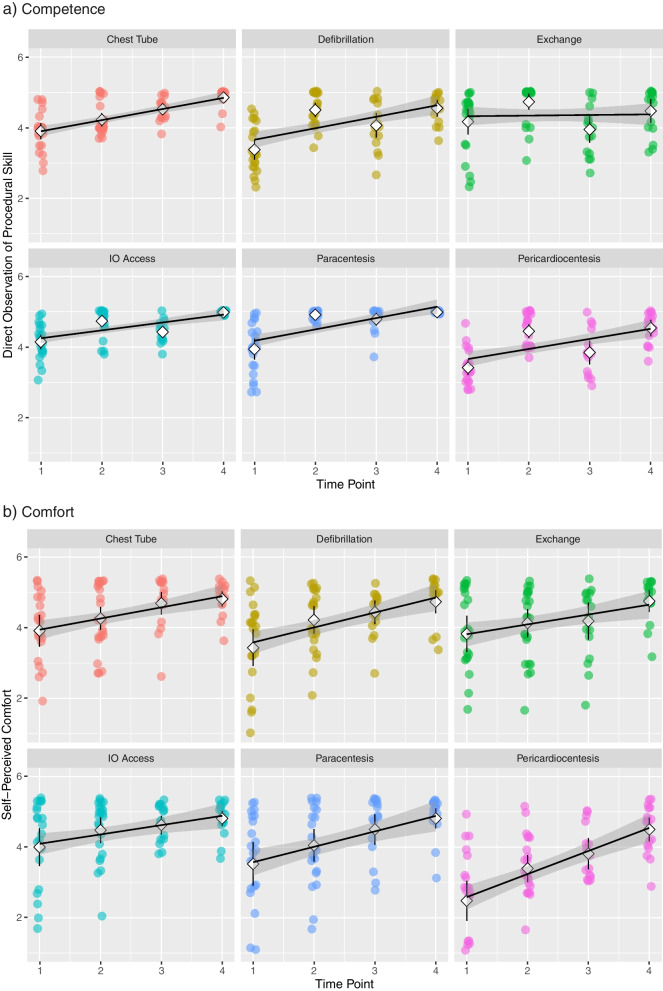
Each skill over 4 time points, self-assessment via Likert Scale **a**) Self-Perceived Competence **b**) Comfort

Self-perceived competence for all six procedures increased over the four time points by 0.51 (95% CI 0.43 – 0.59) combined, with the steepest curve for Pericardiocentesis and the flattest for Exchange Transfusion (Fig. 2a).

**Fig. 3:**
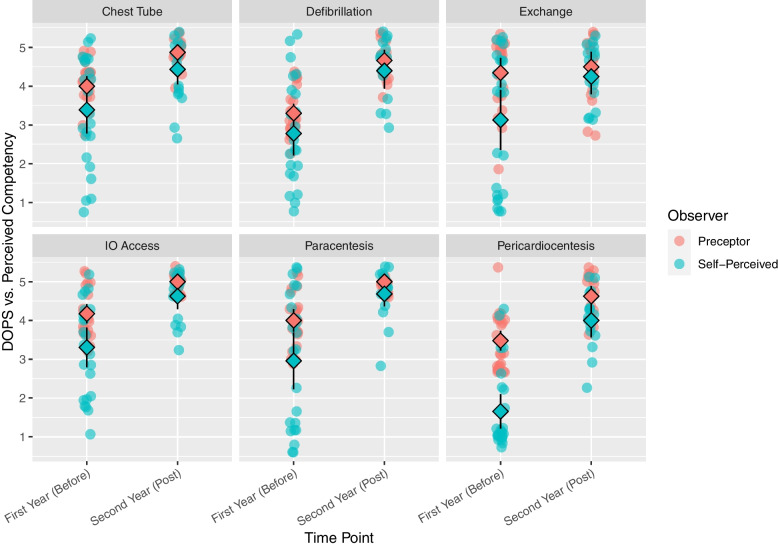
DOPS (Direct Observation of Procedural Skill) compared to self-perceived competence for 6 procedural skills pre- and post-intervention (Time points 1 vs 4)

Self-perceived comfort for all six procedures increased over the four time points by 0.44 (95%CI 0.36 – 0.53) combined, with again Pericardiocentesis being the steepest, the other skills were variable (Fig. 2b). When comparing DOPS vs self-perceived competence, all skills were judged as better by the preceptor, compared to self-assessment, with some discrepancy, most notably for Pericardiocentesis and Exchange Transfusion (Fig. 3).

Cognitive Knowledge across all six procedures increased over the four time points by 0.12 (95% CI 0.06 – 0.18) combined, with the steepest linear trend for Defibrillation and Paracentesis and the flattest for Chest tube insertion and IO access.

## Discussion

Our simulation training “Advanced Neonatal Procedural Skills Day” improved trainee performance in six rarely performed but potentially life-saving clinical skills (Fig. 1). Cognitive knowledge, self-perceived competence and comfort all increased, with most improvement seen in the DOPS scores which increased after first intervention. In the order of skill increase most improvement was seen for Defibrillation, Chest Tube insertion, Paracentesis, Pericardiocentesis, and IO access, while Exchange transfusion skills remained stagnant. Most at-risk for skill decay was Exchange Transfusion, followed by IO access, Pericardiocentesis, and Defibrillation. This may directly inform curricula development aiming to teach these advanced neonatal skills, especially moving towards CBD where simulation is the most viable route for such teaching.

Simulation has been used widely to improve neonatal skill acquisition in boot-camp-like workshops such as this one, [31, 32] as overall the clinical experiences cannot consistently meet training demands [11]. The most common skill targeted by neonatal care providers in the literature has been airway management and intubation which has been shown improvements following simulation training [33]. In a workshop similar to ours for 16 pediatric residents, chest tube insertion similarly was found to be the most improved skill, among lumbar puncture, intubation, bag-valve ventilation, pleural tap and central line insertion [13]. They did not include Defibrillation and the follow-up was restricted to 1 week later. Our study is the first to assess systematically not only skill acquisition with a workshop, but also skill retention and decay over a longer time frame of 9–12 months. As we transition to CBD in pediatric and neonatal education, regular formative and summative assessment of learners will become commonplace and necessary. Rare procedural skills are nearly impossible to assess in the workplace without compromising patient safety. Simulation can help address learning needs on-demand and in a supervised environment; it can serve to expand gaps imposed by the infrequent occurrence of certain conditions and/or complications.

Especially Chest Tube insertion and Defibrillation, followed by the 2 ultrasound-guided skills Pericardiocentesis and Paracentesis and IO showed steep increases with instruction. Self-perceived competence and comfort increased congruently with DOPS assessments suggesting a “true” increase in competence and appropriate self-gauge. Defibrillation stood out due to the highest risk of skill decay which may be explained by the intricate nature of decision-making and perceived high stakes under pressure. CMGs seemed to perform better in this particular skill, compared to IMGs, which may be explained by the frequent practice of Pediatric Advanced Life Support in the CMG group. Particularly for an IMG trainee this skill may be worth consolidating with several instructional sessions and built-in repetition.

Pericardiocentesis stood out as the having the most discrepancy between what the trainees thought they could do versus the better preceptor assessment. It also had the most increase in self-assessed competence and comfort with instruction which may speak to the fact that it is an extremely rarely encountered skill, and intervention is simple enough if practiced before. Instruction here may be sufficient annually, or even biannually. Generally, trainees underestimated their preceptor-assessed skill which may be reflecting professional modesty, or simply discomfort.

Exchange Transfusion stood out as a skill that showed the least impact of the intervention, and also no impact on either self-perceived competence, nor comfort, during the observed time frame, but conversely showed the most skill decay. This skill also showed the most discrepancy between DOPS vs self-assessments (Fig. 3), indicating that participants performed better than they thought. The reason behind this may only be speculated on: This is a generally nursing-driven skill, and few exam questions have been dedicated to it. Motivation may have been reduced, which is a known important factor required for skill acquisition [34]. Simulation may also not be the best way to teach this particular skill, as there are fewer manual skills involved, after the tubing has been assembled, compared to the other skills.

With the inauguration of this annual educational activity, educators and trainees acknowledged the impact, particularly for CBD. We chose to continue the annual skills day beyond this research study. With our results we can give valuable recommendation as to frequency of educational intervention and specific things to keep in mind when dealing with IMGs vs CMGs. Our results may be generalizable to similar trainees but need to be ascertained in other cohorts.

### Strengths and limitations

Our cohort study assessed a group of learners over a 12-month time period with 70% completing both workshops. Assessment was consistent, with valid pre-identified tools with the same 1–2 instructors for each intervention. We could not standardize to pre-intervention skill level or exposure to these skills in clinical environment, due to small sample size. There might have been some skills encountered outside of the intervention. Also there was no prior training for preceptors before the course, nor were we able to calibrate the preceptor assessments. Cognitive skill assessment may have been limited by lack of effort by the trainees due to low stakes and no specific review.

## Conclusions

The simulation-based intervention “Advanced Neonatal Procedural Skills Day” increased preceptor-assessed directly observed procedural skills for all skills examined, except exchange transfusion. Skill decay affected all skills after 9–12 months except Intraosseous and Paracentesis with most decrease in Exchange Transfusion. Exchange Transfusion seemed less amenable to teaching via simulation. Chest Tube insertion and Defibrillation may be best suited for deliberate reminder sessions, especially for IMG trainees. Pericardiocentesis showed the most increase in self-assessed competence and comfort levels and may be suited well by teaching once during training. The effect of a booster session was less than the first intervention, but the final scores were higher than pre-intervention. Trainees underestimated their own skills consistently compared to preceptors’ assessment.

## Supplementary Information

**Additional file 1:** **Appendix A.** Task trainers for Pericardiocentesis and Paracentesis, locally designed by AIS and Norma Oliver, RN.

**Additional file 2:** **Appendix B.** Sample Welcome Letter with Course preparation and links.

**Additional file 3:** **Appendix C.** Timeline.

**Additional file 4:** **Appendix D.** Take-Home Summary.

**Additional file 5:** **Appendix E.** Sample DOPS.

## Data Availability

Raw data and excel sheets are available from the corresponding author upon request.

## Abbreviations

CBD: Competency by design
CMG: Canadian medical graduate
DOPS: Direct observation of procedural skills
IMG: International medical graduate
IO: Intra-osseus
MOC: Maintenance of certification
NICU: Neonatal intensive care unit
NPM: Neonatal perinatal medicine
NRP: Neonatal resuscitation program
OHMES: Office of the health medical education scholarship
PD: Program director
RCPSC: Royal college of physicians and surgeons of Canada
