# Supplementary figures and images for: Advanced neonatal procedural skills: a simulation-based workshop: impact and skill decay
Source: BMC Med Educ. 2023 Jan 13;23:26. doi: 10.1186/s12909-023-04000-1 (PMC9837896; doi:10.1186/s12909-023-04000-1)

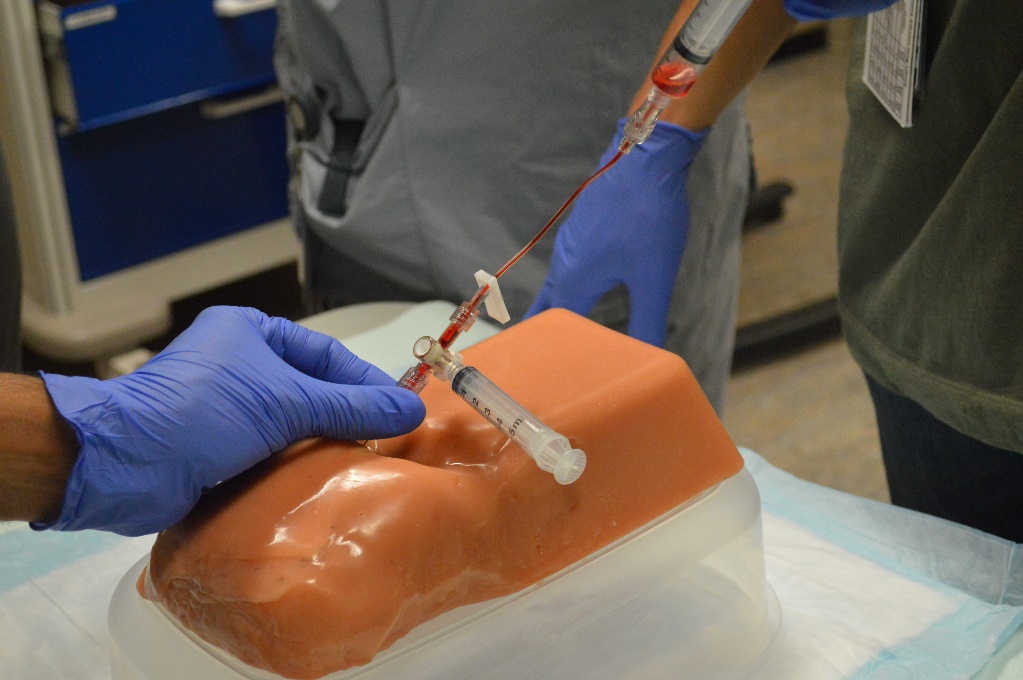
 **Pericardiocentesis Task Trainer**


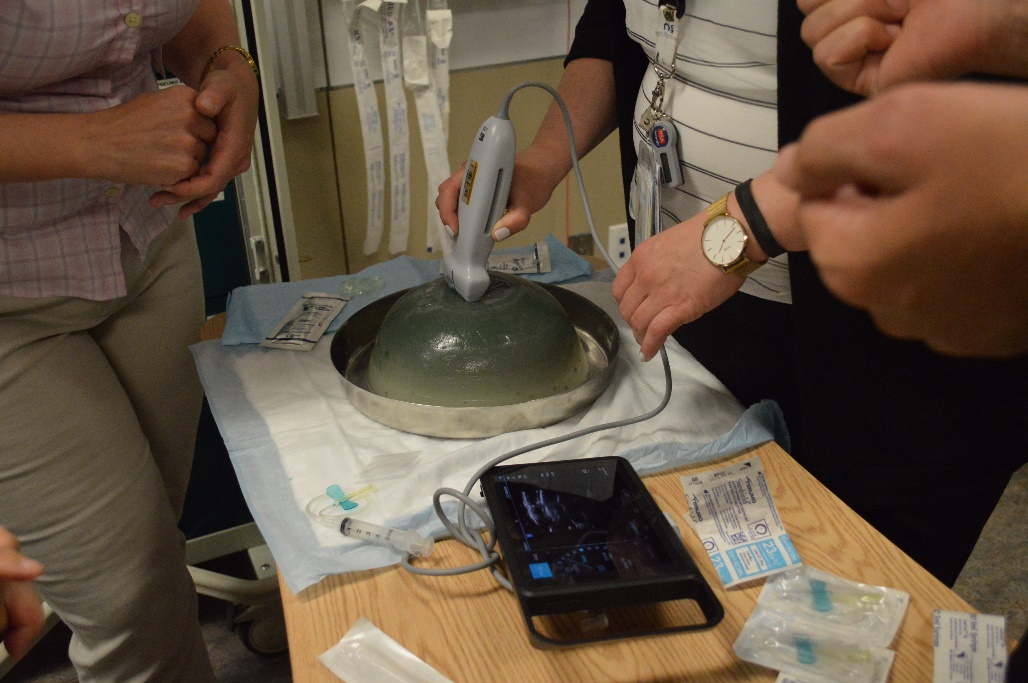
 **Paracentesis Task Trainer**

2.c

3.b

Supplement: Supplementary file 1 — Additional file 1: Appendix A. Task trainers for Pericardiocentesis and Paracentesis, locally designed by AIS and Norma Oliver, RN. [file 12909_2023_4000_MOESM1_ESM.docx]

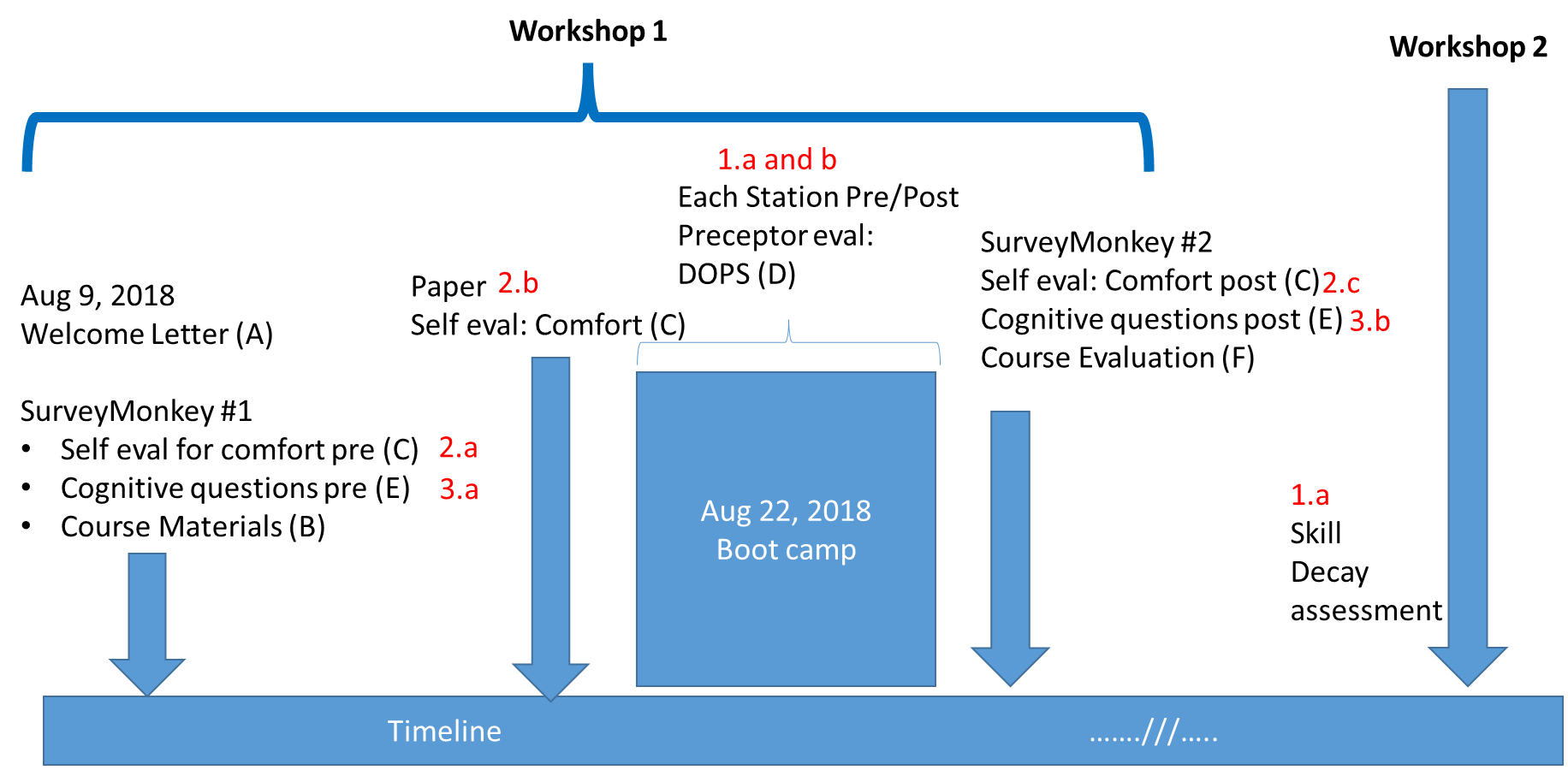

Supplement: Supplementary file 3 — Additional file 3: Appendix C. Timeline. [file 12909_2023_4000_MOESM3_ESM.docx]
