# Supplementary material for: Advanced neonatal procedural skills: a simulation-based workshop: impact and skill decay
Source: BMC Med Educ. 2023 Jan 13;23:26. doi: 10.1186/s12909-023-04000-1 (PMC9837896; doi:10.1186/s12909-023-04000-1)
Supplement: Supplementary file 2 — Additional file 2: Appendix B. Sample Welcome Letter with Course preparation and links. [file 12909_2023_4000_MOESM2_ESM.docx]

**Advanced neonatal procedural skills –simulation-based boot camp**

**Aug 22, 2018, KidSim Alberta Children’s Hospital**

Amelie Stritzke, Prashanth Murthy, Elsa Fiedrich, Michael Assaad, Alixe Howlett, Adam Cheng, Harish Amin

**BACKGROUND**

**Background:**

Procedural competence is defined as “the ability to perform a procedure independently, without supervision, with a high likelihood of successful completion.” (Gaies, 2007) Neonatal Perinatal Medicine (NPM) trainees must be competent in 19 procedural skills as per the Royal College of Canada (RCPSC) before completing a two year program. Currently, there is no standardized curriculum, defined competence, or mandated recording for these skills. Clinical opportunity is rare, particularly in less eoften performed neonatal procedures. No formal evaluation of which skills require more training exists.

**Needs assessment:**

A cross-sectional confidential electronic survey was completed by 47 trainees (68% response rate) by Year 1 (>6 mos) and 2 (<28 mos) NPM trainees in 13 Canadian NPM programs (2012- 2013). Less commonly performed procedures had lesser competence scores such as pericardiocentesis (0.45), and paracentesis (1.00), exchange transfusion (3.1) and chest tube insertion.

**Methods:**

- Boot camp in KidSim™ lab, Alberta Children’s Hospital
- AHD: 12:30-16:00 August 22, 2018
- Learners: 18 (13 NPM fellows and 5 senior pediatric residents)
- Instructors: 5 Neonatologists, 2 PALS nurses, 1 neonatal NP
- Review indications/contraindications and potential complications before boot camp
- Supported by pediatric residency program director Dr. DiBartolo and NPM fellowship program director Dr. Kamaluddeen

| **Monday August 13, 2018** |
| --- |
| - Course material sent out to review - Informed consent sent out to read, fill out and send back or bring day of - Please ensure all links for each station materials are reviewed before attending academic half day |

**Stations:**

- 6 stations
- 3 rooms reserved at KidSim™
- Groups of 3 participants each
- 30 min each
- 1min individual Pre-assessment per room (DOPS pre)

**ROOM 1**

| **Station 1** | **IO access** |
| --- | --- |
| Facilitator(s) | PALS nurse (Paula Espinoza) and neonatologist (Elsa Fiedrich) |
| Equipment | IO drills x 2, bones x 6, iv fluid and tubing |
| Preparation | NRP video on IO in neonates  <https://www.youtube.com/watch?v=mpnroZi8t0A> |
|  |  |
| **Station 2** | **Defibrillation and external pacing** |
| Facilitator(s) | PALS nurse (Connie Abrey) and neonatologist (Alex Howlett) |
| Equipment | Doll, defibrillator with pads, PALS guideline |
| Preparation | <https://www.youtube.com/watch?v=1fjmyog37Fo>  <https://www.youtube.com/watch?v=XaC1diqELHw> |

**ROOM 2**

| **Station 3** | **Paracentesis (ultrasound guided)** |
| --- | --- |
| Facilitator(s) | Neonatologist (Amelie Stritzke) |
| Equipment | Task trainer; ultrasound machine, gel, cleaning wipes, gloves |
| Preparation | Video on paracentesis  <https://www.youtube.com/watch?v=wKYWhutqzyg> |
|  |  |
| **Station 4** | **Pericardiocentesis (ultrasound guided)** |
| Facilitator(s) | Neonatologist (Prashanth Murthy) |
| Equipment | Task trainer as per Zerth 2012, ultrasound machine, gel, cleaning wipes, gloves |
| Preparation | Video on pericardiocentesis  <https://www.youtube.com/watch?v=wKYWhutqzyg> |

**ROOM 3**

| **Station 5** | **Chest tube insertion** |
| --- | --- |
| Facilitator(s) | Neonatologist (Harish Amin) |
| Equipment | 6 chickens prepared with chlorhexidine, gloves, big plastic cover for bed; chest tube insertion kit x 8 |
| Preparation | <https://www.youtube.com/watch?v=PgBa-sRbK7w> |
|  |  |
|  | **ROOM 4** |
| **Station 6** | **Exchange transfusion** |
| Facilitator(s) | Neonatal Nurse Practitioner (Jennifer Orton) |
| Equipment | Doll, iv bags and set-up, fake blood, umbilical stumps, iv pumps and fluid, ECG for complications, CPG from NICU |
| Preparation | Videos on set-up and exchange transfusion:  Setting up Neonatal Exchange Transfusion - 2 lines  <https://www.youtube.com/watch?v=eE9LMDpaPRw>  Setting up Neonatal Exchange transfusion – UVC only (1 venous line only)  <https://www.youtube.com/watch?v=S1GrYSFmRig>  Long but good Utube on Exchange Transfusion  <https://learn.transfusion.com.au/course/view.php?id=402> |

| **Wednesday Aug 22, 2018:** Schedule | |
| --- | --- |
| 12:30 | - Welcome and Course Overview - Pre-Course Survey and Quizzes - Informed consent form to allow us to analyze the gathered data (if not already returned) |
| 13:00 – 16:00 | - 15 participants divided into 6 groups of 2-3 people each (mixed residents with fellows) - 30 mins per station |
| 16:00 | - Course evaluation - Post-intervention survey and wrap-up |

**Assessment:**

1. Preceptor assessment pre and post intervention for each station and each participant, Direct Observation of Procedural Skill (DOPS)
2. Via Likert scale self-assessment pre and after intervention, how comfortable are you with the procedure on a scale of 1-5 and how readily would you attempt it in a clinical setting if prompted, 1-5?
3. Plan is to repeat the assessment without the teaching component in a second assessment via same skill station one year after; without guidance to look at retention of skill/skill decay.

**Ethics:** REB18-1079

**Outcome/Impact**:

Before CBD implementation show that simulation based training is adequate to address shortage of opportunity for rare procedural skills in NICU. May be used in training, formative and summative assessments.

**Participants - aim 15 (max 18):**
