# Supplementary material for: Advanced neonatal procedural skills: a simulation-based workshop: impact and skill decay
Source: BMC Med Educ. 2023 Jan 13;23:26. doi: 10.1186/s12909-023-04000-1 (PMC9837896; doi:10.1186/s12909-023-04000-1)
Supplement: Supplementary file 4 — Additional file 4: Appendix D. Take-Home Summary. [file 12909_2023_4000_MOESM4_ESM.pdf]

Cardioversion and Defibrillation

Indications

Cardioversion (Synchronized)	Defibrillation (Asynchronised)
Unstable SVT, VT, AF, A. flutter, or stable but not responding to other measures	Pulseless arrest with shockable rhythm; VT, VF

Paddle Placement:

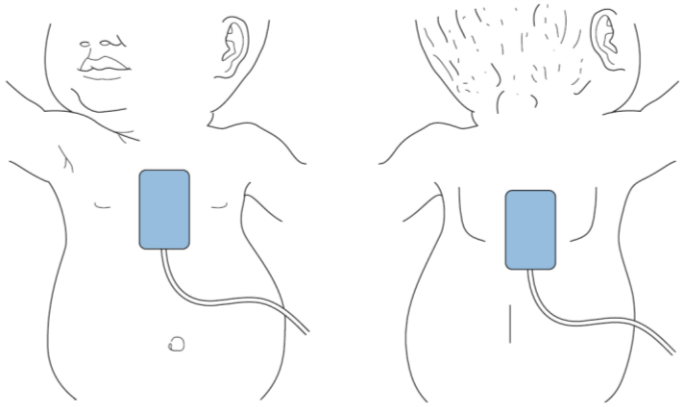

Remember to:

Protect airway

Sedate if possible

Wipe the chest clean (e.g. remove echo gel)

Pads not in contact with one another

Energy:

Cardioversion	Defibrillation
0.5 J/kg → 1 J/kg → 2 J/kg	2 J/kg → 4 J/kg<br>*Cont. CPR+ medications

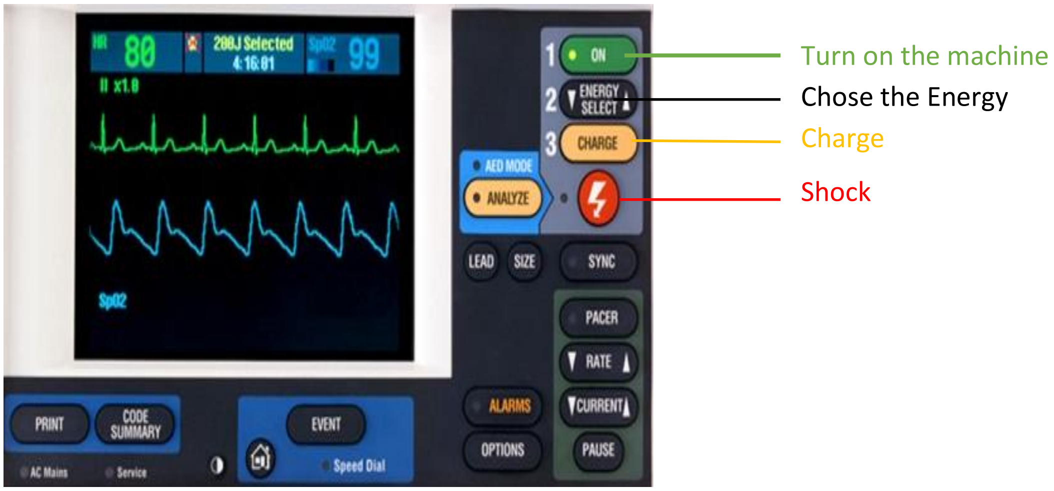

Pacing

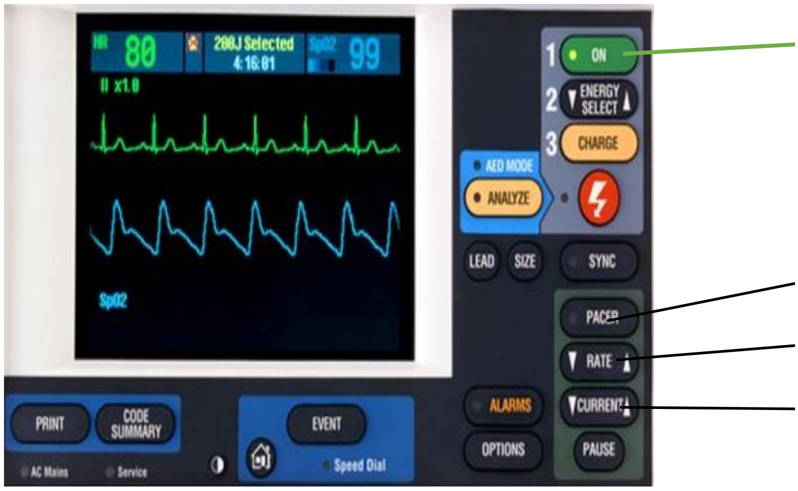

Turn on the machine

Turn on Pacing

Set the rate (Start 100)

Increase the current to capture all spikes

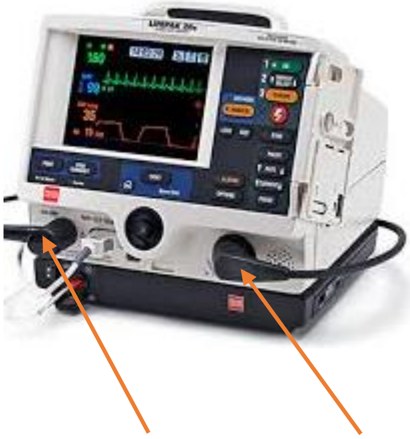

Make sure the pacing and monitor cables are both connected

Chest Tube

Indications	Air	Fluid
Emergency relief site	Mid-Clavicular 2nd ICS	–
CT site	4th or 5th ICS between ant. & mid. Axillary line (One to two ICS below the nipple)	
CT direction	Anteriorly & up	Posteriorly & down

Practical points

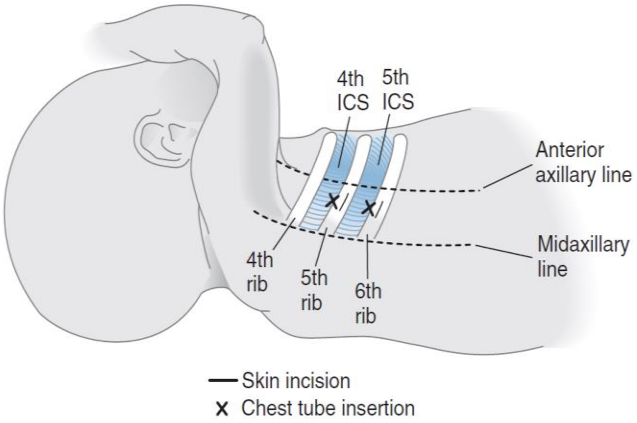

Incision is over the rib and insertion is just above the rib to avoid damage to the IC neurovascular bundle

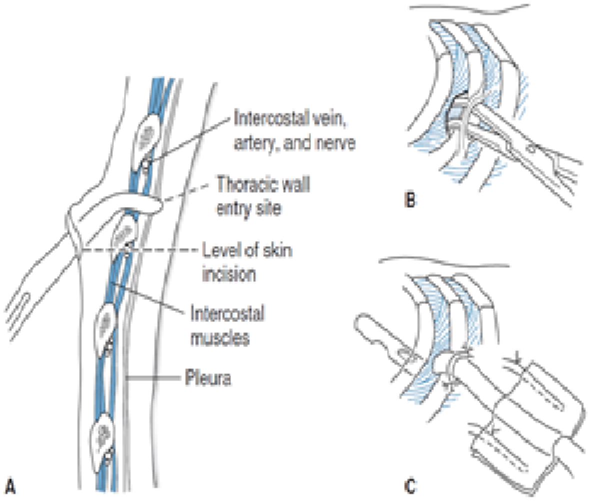

Introduce a closed hemostat to puncture the pleura

Bevel up for air and down for fluid helps in guiding the CT direction

Open the hemostat & introduce the CT through it 2-3 cm for PT & 3-4 cm for FT

Paracentesis

Baby position: Supine and slightly turned toward the insertion side

Insertion site: McBurney's point. Choose location after checking the deepest pocket by US

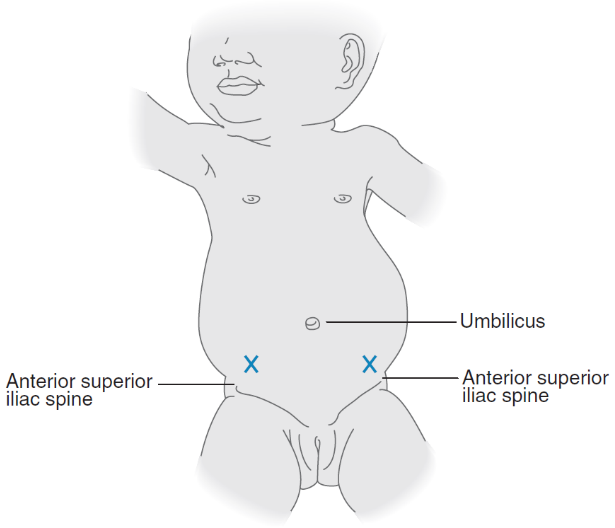

Insert the needle perpendicular to the skin

When it's just under the skin move it by 0.5 cm to create a Z-track

Puncture the abdominal wall 45^0 toward the back

Advance the needle just enough to obtain fluid

Pericardiocentesis

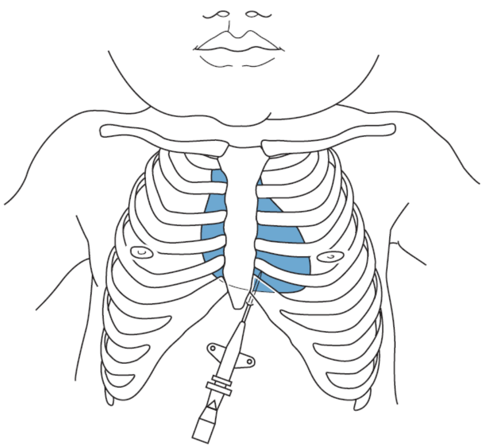

Insert the needle 0.5 cm just below and to the left of the xiphoid

Insert at 30^0 toward the left mid-clavicular line

Advance just enough to get air/fluid

ECG changes indicate the needle is touching the myocardium and needs to be pulled back

EZ-IO (ideal >3kg, min 2kg)

Sites

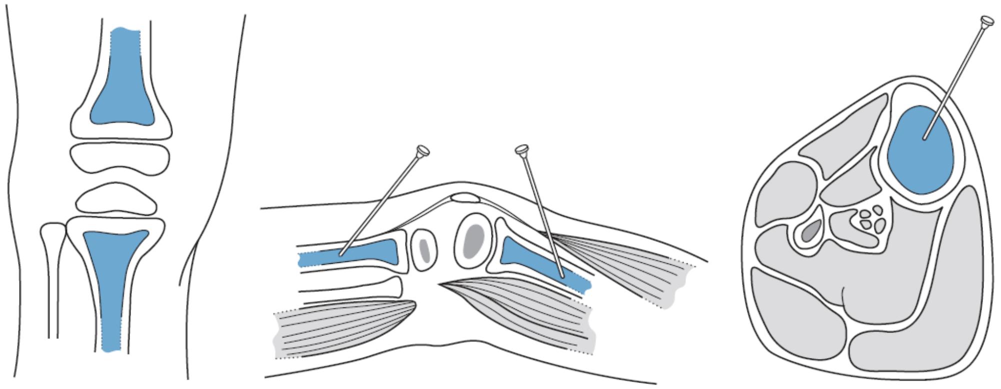

Proximal tibia: 1-2 cm below & medial to the tibial tuberosity (Flat anteromedial surface)

Distal femur: Ant. Midline 1-3 cm above the external condyles

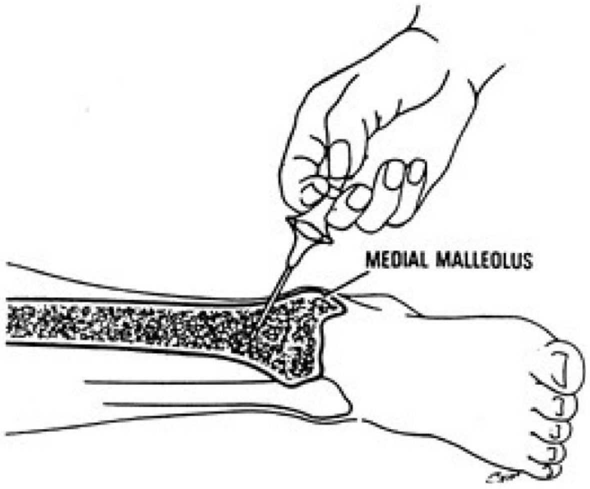

Distal tibia: medial surface just proximal to the medial malleolus

Made by Haytham Eid, July 2019
