# Supplementary material for: Advanced neonatal procedural skills: a simulation-based workshop: impact and skill decay
Source: BMC Med Educ. 2023 Jan 13;23:26. doi: 10.1186/s12909-023-04000-1 (PMC9837896; doi:10.1186/s12909-023-04000-1)
Supplement: Supplementary file 5 — Additional file 5: Appendix E. Sample DOPS. [file 12909_2023_4000_MOESM5_ESM.docx]

**SECTION OF NEONATOLOGY**

**Advanced neonatal procedural skills –simulation-based boot camp**

**July 3, 2019**

Station 1: IO access in Neonates

Participant Number:

PRE-Post Intervention assessment by facilitator name:

|  | | **2** | **3** | **4** | **5** |
| --- | --- | --- | --- | --- | --- |
| **Indication** | |  |  |  |  |
| **Contraindication (if any)** | |  |  |  |  |
| **Informed Consent** | |  |  |  |  |
| **Appropriate Preparation (pre-procedure)** | |  |  |  |  |
| **Technical ability** | |  |  |  |  |
| **Aseptic technique** | |  |  |  |  |
| **Seeks help where appropriate** | |  |  |  |  |
| **Awareness of complications** | |  |  |  |  |
| **Post procedure management** | |  |  |  |  |
| **Communication skills** | |  |  |  |  |
| **Overall ability** | |  |  |  |  |
| ****** |  | | | | |
| **2** | **I had to walk him/her through it. Able to perform task but required constant direction** | | | | |
| **3** | **I had to help a little. Required intermittent direction** | | | | |
| **4** | **I was there just in case. Still required supervision for safe practice, unaware of risks.** | | | | |
| **5** | **I did not have to be there. Complete independence, understands risks and performs safely. Practice ready.** | | | | |

*Adapted from Barton JR, 2012 **Adapted from Gofton WT, 2012
